# Synergistic Effect of NaCl Pretreatment and PVP on Browning Suppression and Callus Induction from Petal Explants of *Paeonia Lactiflora* Pall. ‘Festival Maxima’

**DOI:** 10.3390/plants9030346

**Published:** 2020-03-09

**Authors:** Xuan Cai, Hao Wei, Chen Liu, Xiuxia Ren, Luc The Thi, Byoung Ryong Jeong

**Affiliations:** 1 Institute of Agriculture and Life Science, Gyeongsang National University, Jinju 52828, Korea; luckilyxuan@gmail.com; 2 School of Nuclear Technology and Chemistry & Biology, Hubei University of Science and Technology, Xianning 437100, Hubei, China; 3 Division of Applied Life Science (BK21 Plus Program), Graduate School, Gyeongsang National University, Jinju 52828, Korea; oahiew@gmail.com (H.W.); liuchen215@gmail.com (C.L.); renxiuxia@caas.cn (X.R.); thitheluc.la@gmail.com (L.T.T.); 4 Research Institute of Life Science, Gyeongsang National University, Jinju 52828, Korea

**Keywords:** Tissue culture, herbaceous peony, phenolic, phenylalanin ammonia-lyase (PAL), polyphenol oxidase (PPO)

## Abstract

Browning is prevalent in tissue cultures of *Paeonia lactiflora* Pall. (herbaceous peony), and severely affects and restricts the growth and differentiation of the explants. In this study, dipping excised explants in a sodium chloride (NaCl) solution as a pretreatment, adding polyvinyl pyrrolidone (PVP) to the culture medium, storing planted explants at 4 °C for 24 h, and transferring planted explants to a new medium after 24 h were considered as browning-suppression methods in tissue cultures of herbaceous peony ‘Festival Maxima’. The treated petal explants were cultured in a culture room with a 16-hour photoperiod, 25 °C temperature, and 80% relative humidity in darkness for 4 to 8 weeks. The results demonstrated that dipping excised explants in a 0.5 g·L^−1^ NaCl solution, adding 0.5 g·L^−1^ PVP to the medium, storing planted explants at 4 °C for 24 h, and transferring planted explants to the same fresh medium after 24 h could effectively inhibit browning. Adding PVP to the medium led to the greatest browning suppression percentage of 95%. Storing planted explants at 4 °C for 24 h reduced the effectiveness of other treatments in suppressing browning. After 8 weeks, dipping excised explants in a NaCl solution resulted in the highest callus induction percentage of 75%, while storing explants at 4 °C for 24 h suppressed callus formation. It was observed in all treatments that decreases in browning was accompanied with higher levels of phenols and lower activities of phenylalanine ammonia-lyase (PAL) and polyphenoloxidase (PPO). Overall, the results suggest that dipping in a NaCl solution was effective in alleviating the browning issues of herbaceous peony tissue cultures, and had positive synergistic effects with PVP on browning suppression and callus induction.

## 1. Introduction

Herbaceous peony (*Paeonia lactiflora* Pall.) is a popular flower in Korea due to its attractive appearance, fragrance, large flowers, a wide range of colors, and high ornamental value. It is also one of the most famous and traditional flowers in China, where people have cultivated herbaceous peony for more than 3900 years [1]. Herbaceous peony also has a laudatory name, “the queen of flowers.” It is also frequently has been used in Chinese medicine [2].

Herbaceous peonies are generally propagated by seed or by division, cuttage, grafting, and layering [3]. However, these conventional propagation methods can only be carried out in the vegetative season and require a considerable amount of propagation materials [3,4]. In vitro culture is a very effective means to propagate and preserve the germplasm through the mass clonal multiplication. In vitro culture has many advantages, including a short reproductive cycle, high propagation coefficients, and ease of mass production [5]. Therefore, the use of tissue culture to solve the problems associated with conventional propagation methods is attractive in the industrialization and commercialization of herbaceous peonies. However, browning is prevalent in tissue cultures of herbaceous peonies, which severely affects and restricts the growth and differentiation of the explants in vitro.

Browning in plant tissue culture refers to a phenomenon in which the explants release brown substances or phenolics to the medium from its own tissues in the course of dedifferentiation and/or re-differentiation [6,7]. Phenolics have been confirmed to be generated by elevated activity of polyphenol oxidase (PPO) [8]. In addition, the phenylalanine ammonia lyase (PAL) converts phenylalanine to free phenolic substrates for PPO [9]. The PPO and PAL have been reported to be involved in the tissue browning of many species, such as apples [10], pineapples [11], sweet potatoes [12], pears [13,14], and cabbage [15].

Browning is affected by different factors including pretreatments, medium, temperature, and subculture frequency [16]. It has been shown that charcoal can adsorb phenols and thus deprived enzymes of substrates needed for generating the polymers that induce browning [17,18]. Other practices removing phenols or reducing their accumulation in the culture media, such as transferring to a fresh medium or adding polyvinylpyrrolidone (PVP) to the medium, have also alleviated the problem [19]. NaCl was an inhibitor of PPO activity in peach [20]. PPO activity could be inhibited by NaCl as well as ascorbic acid and citric acid [21]. Low temperature storage was reported to increase the PAL activity in walnut [22].

Studies related to browning in herbaceous peonies have mainly focused on the explant materials, basal media, anti-browning agents, culture conditions, and explant pretreatments. In underground buds of *P. lactiflora* ‘Zhong Sheng Fen’, the occurrence of browning was closely related to the sampling time and explant type [23]. It was shown that PVP was the best anti-browning agent for *P. suffruticosa* ‘Luo Yang Hong’ [24]., followed by ascorbic acid (5–15 mg·L^−1^), while Na_2_S_2_O_3_ at 500–1500 mg·L^−1^ was the least effective. The use of the half-strength WPM (Woody Plant medium) medium containing 1000 mg·L^−1^ PVP and 2 mg·L^−1^ Phytagel during the subculture of *P. suffruticosa* ‘Feng Dan’ explants (leaves and petioles) resulted in reduced levels of browning [25]. Adding BA (Benzylaminopurine) (0.5 or 1 mg·L^−1^) exacerbated browning during culture initiation, while changing the concentration of BA and NAA (1-naphthlcetic acid) had no significant impacts on browning [26]. Zhao and Yu [27] used stems, petioles, and leaves of herbaceous peony (*P. lactiflora*) ‘Tao Hua Fei Xue’ to induce callus formation and found that browning was the most serious in the leaf explants.

In vitro culture, petal is reported as an important explant. Petal has been reported that it exhibited the highest frequencies of shoot organoenesis and mean number of shoots per explant in chrysanthemum [28]. For herbaceous peonies petal, studies have mainly focused on postharvest [29,30] and effecting components analysis [31,32]. Still studies to date are limited on browning inhibition of herbaceous peony petals in tissue culture. In this study, NaCl pretreatment, storage at cold temperature, transfer to a new medium, and PVP addition to the medium were studied to compare their effectiveness on the browning suppression and callus induction from the petal explants. The total phenol content, and activities of PPO and PAL were also measured to determine how they were related to browning under the above conditions. The contamination and callus induction percentages of the petal explants were also recorded under these treatments.

## 2. Materials and Methods

### 2.1. Plant Materials

Cut flowers of *Paeonia lactiflora* Pall. ‘Festival Maxima’ with tight flower buds were purchased from the World Flower Company (Jincheon, Republic of Korea). The cut flowers were maintained at 4 °C for up to 30 days and used for experiments. Then, the flower buds were thoroughly washed with running tap water for 3 h, and subsequently washed with 70% (v/v) ethanol (Yakuri Pure Chemicals, Kyoto, Japan) for 2 minutes, and 1.5% (v/v) sodium hypochlorite (NaOCl) (Yakuri Pure Chemicals, Kyoto, Japan) for 15 minutes with a couple of drops of Tween 20 (Yakuri Pure 103 Chemicals, Kyoto, Japan), followed by 3–4 washes with disinfected distilled water. Outer scales or petals were removed in a laminar flow hood (Daejin, Incheon, Republic of Korea) and the remaining buds were washed again with 70% ethanol for 1 minute, 1.5% NaOCl for 10 minutes, and 0.1% HgCl_2_ for 15 minutes, followed by 3–4 washes with disinfected distilled water.

### 2.2. Tissue Culture

The petal explants taken from the remaining flower buds were cut into 1–2 cm long sections. They were planted to the medium immediately after each treatment. A 25 mL Anderson’s rhododendron medium [33] (Duchefa, Haarlem, The Netherlands) solidified with 0.75–0.80% (w/v) agar (Yakuri Pure 103 Chemicals, Kyoto, Japan) and supplemented with 3% (w/v) sucrose (Yakuri Pure 103 Chemicals, Kyoto, Japan) and plant growth regulators (PGRs) (Yakuri Pure 103 Chemicals, Kyoto, Japan) (in mg·L^−1^ at 2.0 thidiazuron (TDZ), 0.5 2iP, 2.0 2,4-D, and 5.0 GA_3_ was dispensed in each 90 cm petri dish (10090, SPL, Pocheon, Republic of Korea). All explants were cultured at 25 °C in darkness for 4 weeks and then were exposed to fluorescent light (60–100 mol m^−2^·s^−1^ photosynthetic photon flux density (PPFD)) with a 16-hour light and 8-hour dark photoperiod. Cultures were observed at 0 and 1 day, and 1, 2, 3, and 4 weeks after the initiation of culture, and percentages of contamination and callus induction were measured.

### 2.3. Treatments

Dipping in a NaCl (Yakuri Pure 103 Chemicals, Kyoto, Japan) solution, supplementing the medium with polyvinyl pyrrolidone (PVP) (Yakuri Pure 103 Chemicals, Kyoto, Japan), low temperature storage, and transfer of the explants to a new medium were the four methods used in this study. The details on the treatment combinations are listed in Table 1, and denoted as follows:

Na means dipping petals in a 0.5 g·L^−1^ NaCl solution before planting;

P means addition of 0.5 g·L^−1^ PVP to the medium;

L means storage at 4 °C for 24 h after planting;

NT means no transfer;

T means transfer to a new medium after 24 h of planting.

Treatments were set into 4 groups: 

a. group contains NT, T, and PNT; 

b. group contains NaNT, NaT, and NaPNT; 

c. group contains LNT, LT and LPNT;

d. group contains NaLNT, NaLT, and NaLPNT. 

### 2.4. Browning Evaluation

The browning of the cut edges of the petal explants was visually evaluated. The percentage of explants with browning was evaluated at 0 and 1 day, and 1, 2, 3, and 4 weeks after the initiation of culture. The extent of browning was denoted by S0 (no browning), S1 (slight browning), S2 (definite browning), and S3 (extreme browning).

### 2.5. Total Phenolic Contents

A total of 3 mg of tissue was homogenized in the mortar with 1.5 mL distilled water. The homogenate was centrifuged for 15 minutes at 12,000 *x*g. The supernatant was carefully transferred to a 1.5 mL centrifuge tube (10090, SPL, Pocheon, Republic of Korea). The Folin-Ciocalteu procedure was followed using a commercial reagent (Yakuri Pure 103 Chemicals, Kyoto, Japan) [34]. Then 0.4 mL of the supernatant was added into a tube containing 3.6 mL distilled water. After being shaken, a 0.4 mL Folin-Ciocalteu reagent was added. Following another shake, a 7% (w/v) Na_2_CO_3_ solution was added after 5 minutes. After fully mixing, distilled water was added to the mixture to make the total volume 10 mL. After 90 minutes at room temperature avoiding light, absorbances at 765 nm were recorded by UV-spectrophotometer (Libra S22, Biochrom Ltd., Cambridge, UK). The phenol standards were prepared by dissolving 10 mg of gallic acid (Yakuri Pure 103 Chemicals, Kyoto, Japan) in water and diluting it to 50 mL. The Folin-Ciocalteu protocol was applied to the solution containing 0–0.06 mg·mL^−1^ gallic acid to construct the standard curve.

### 2.6. Enzyme Extraction and Assay 

#### 2.6.1. Polyphenol Oxidase (PPO)

Referred to Hisaminata’s method [35], a 3 mg of tissue was homogenized in the mortar with a 1.5 mL MacⅡvaine buffer (pH 6.5; a mixture of 0.1 M citric acid (Yakuri Pure 103 Chemicals, Kyoto, Japan) and 0.2 M disodium phosphate (Yakuri Pure 103 Chemicals, Kyoto, Japan)). The homogenate was centrifuged for 15 minutes at 12,000 *x*g. The supernatant was carefully transferred to a 1.5 mL centrifuge tube and used as a crude enzyme of PPO. The activity of PPO was measured spectrophotometrically at 410 nm to detect the decrease of chlorogenic acid (Yakuri Pure 103 Chemicals, Kyoto, Japan) as a substrate. The reaction solution consisted of 2 mL of MacⅡvaine buffer (pH 6.0), 0.5 mL of 0.1 mol·L^−1^ catechol (Yakuri Pure 103 Chemicals, Kyoto, Japan), and 0.5 mL of the crude enzyme solution. A total of three 3-minute reads was made with a 15 second recording interval. An increase in the absorbance of 0.001 per minute at 30 °C was defined as one unit.

#### 2.6.2. Phenylalanin Ammonia-Lyase (PAL)

Referred to Hisaminata’s method [35], a 3 mg of tissue was homogenized in the mortar with a 1.5 mL 0.2 M boric acid-NaOH (Yakuri Pure 103 Chemicals, Kyoto, Japan) buffer (pH 8.8). The homogenate was centrifuged for 15 minutes at 12,000 *x*g. The supernatant was carefully transferred to a 1.5 mL centrifuge tube and used as a crude enzyme of PAL. The activity of PAL was measured spectrophotometrically at 290 nm to detect the increase in cinnamic acid as a product. The reaction solution consisted of 2.0 mL of distilled water, 1.0 mL of 0.02 mol·L^−1^ L-phenylalanine (Yakuri Pure 103 Chemicals, Kyoto, Japan), and 1.0 mL of the crude enzyme solution. The solution was placed in a 30 °C water bath for 1 h to react after being fully shaken. One unit of PAL activity was defined as the amount of enzyme that produced one micromole of cinnamic acid.

### 2.7. Statistical Analysis

Each treatment was replicated at least four times, and all data were subjected to an analysis of standard deviation (SD) using SAS (SAS Institute, Cary, NC, USA) and to the least significant difference (LSD) test. The significance was set at *p* ≤ 0.05 for comparing the means.

## 3. Results and Discussion

### 3.1. Effects of Treatments on Explant Browning

**Figure 1:**
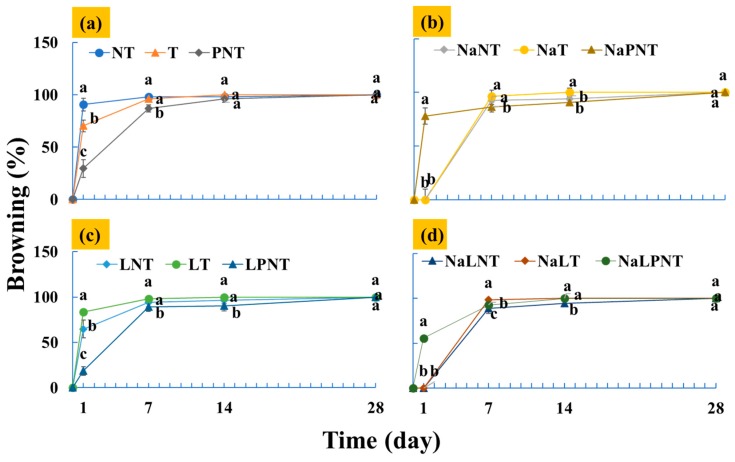
Browning percentage of the petal explants of herbaceous peony ‘Festival Maxima’. The different abbreviations denote as follows: Na, dipping in a 0.5 g·L^−1^ NaCl solution before planting; P, 0.5 g·L^−1^ polyvinyl pyrrolidone (PVP) addition to the medium; L, storage at 4 °C for 24 h after planting; NT, not transferring to a new medium; and T, transferring to a new medium at 24 h after planting. Treatments were set into 4 groups; (**a**), NT, T, and PNT; (**b**), NaNT, NaT, and NaPNT; (**c**), LNT, LT, and LPNT; and (**d**), NaLNT, NaLT, and NaLPNT. Different alphabet letters indicate differences between treatments (ANOVA and Duncan’s test, *p* ≤ 0.05).

Figure 1 shows the effect of treatments on the browning of petal explants of herbaceous peony during the initial stage of tissue culture. Figure 1a compares the effect of transferring the explants to a new medium and adding PVP to the medium on explant browning. The browning percentage increased rapidly to 90.7% at 1 day after culture initiation in the treatment in which the explants were not transferred to a new medium. Transferring to a new medium and adding PVP significantly reduced the browning percentage to 70.4 and 29.6%, respectively. At 1 day after culture initiation, browning appeared in most of the petal explants, and there were no significant differences in the browning percentage of the explants among the three treatments. Similar to those observed in this study, transferring to a fresh medium and adding PVP to the medium were reported to be practical methods to reduce the accumulation of browning compounds [19]. In this study, adding PVP was more effective than transferring to a new medium in reducing the browning, which may be due to the absorption of some browning compounds by PVP.

Figure 1b shows the effects on explant browning of dipping the explants in a 0.5 g·L^−1^ NaCl solution before planting, and a combination of the NaCl pretreatment, PVP addition and transferring to a new medium. At 1 day after culture initiation, there were no browned explants found in the NaNT and NaT, while the browning percentage of in the NaPNT reached to 77.8%. However, there were no significant differences in the browning of the explants among the treatments at 4 week after culture initiation. It was shown that browning could be effectively inhibited by dipping the explants in a NaCl solution. The NaCl has been commonly used to inhibit browning of apples. A strongly pH-dependent PPO inhibitory effect was observed by NaCl [36]. However, when applied in conjunction with PVP, the effectiveness of NaCl in browning inhibition was reduced, which may be due to adsorption by PVP.

Figure 1c shows the effect on explant browning of storing the explants at 4 °C for 24 h after planting, and the combination of low temperature, PVP, and transferring to a new medium. At this low temperature, the browning percentages at 1 day after culture initiation of the explants transferred to a new medium, not transferred to a new medium, and PVP added were 64.8, 84.3, and 18.5%, respectively. Compared to the results shown Figure 1a, the low temperature storage was more effective in inhibiting browning. The low temperature storage has been used to inhibit browning in some vegetables. At 4 week after culture initiation, there were no significant differences in the browning of the explants among treatments.

Figure 1d shows combined effects of NaCl pretreatment, PVP addition, low temperature storage, and transferring to a new medium. As the results in Figure 1b, regardless of whether the explants were transferred to a new medium or not, there were no browning explants observed at 1 day after culture initiation when the explants were dipped in a 0.5 g·L^−1^ NaCl solution before planting and being stored at 4 °C for 24 h. The browning percentage of the explants in the NaLPNT at 1 day after culture initiation was 55.6%, which was higher than that in the LPNT, but lower than that in the NaPNT. It seems that low temperature storage could reduce the negative effect of PVP to NaCl on browning inhibition. At 4 weeks after culture initiation, there were no significant differences in the browning percentage among treatments.

**Figure 2:**
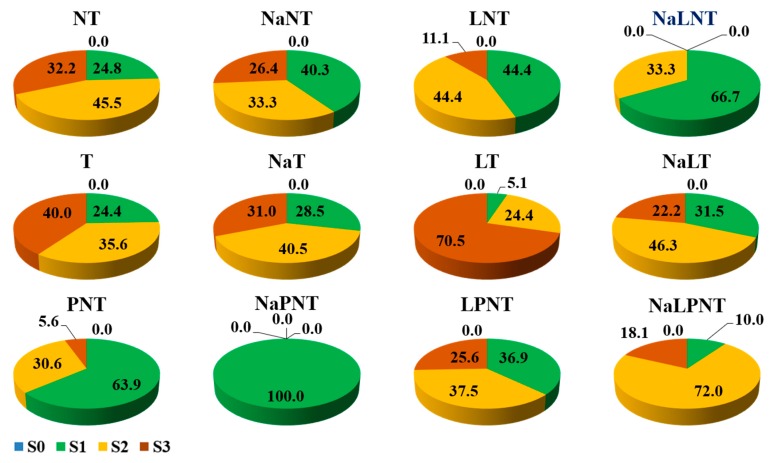
The extent of browning of the petal explants observed at 28 days after culture initiation: S0, no browning; S1, slight browning; S2, definite browning; and S3, extreme browning. Refer to Figure 1 for details on the different denotations.

The effects of treatments on the browning of the explants observed at 4 weeks after culture initiation, graded on a scale of S0 (no browning), S1 (slight browning), S2 (definite browning), and S3 (extreme browning), are shown in Figure 2. For the explants not transferred to a new medium, the treatment of dipping in a 0.5 g·L^−1^ NaCl solution before planting, and the treatment of storing at 4 °C for 24 h after planting significantly delayed browning of the explants. The percentage of the explants at S1 was 40.3% and 44.4% for the above two treatments, NaNT and LNT, respectively. When these two treatments were applied in a combination, the browning inhibition was enhanced to have 66.7% of the explants at S1, 33.3% of them at S2, and none of them at S3 (Figure 2, NALNT). It seems that NaCl solution dipping and low temperature storage were the two most practical methods in suppressing tissue browning [36,37].

When the explants were transferred to a new medium, 24.4%, 35.6%, and 40.0% of the explants were at S1, S2, and S3, respectively (Figure 2, T). Dipping the explants in a 0.5 g·L^−1^ NaCl solution before planting delayed the browning to a S3 level, while storing the explants at 4 °C for 24 h after planting accelerated the browning to a S3 level (Figure 2, NaT and LT). A combined treatment of dipping in a 0.5 g·L^−1^ NaCl solution before planting and storing at 4 °C for 24 h after planting resulted in the most of the explants being at S1 (31.5%) and S2 (46.3%) levels (Figure 2, NaLT).

When PVP was added to the medium, the browning was very effectively suppressed to have 63.9% of the explants at S1 (Figure 2, PNT), a much higher proportion than that of the NT or T. When these explants were dipped in a 0.5 g·L^−1^ NaCl solution before being planted, all explants were at S1 (Figure 2, NaPNT), representing a significant browning inhibition. However, storing the explants at 4 °C for 24 h after planting along with PVP addition to the medium increased the percentage of S2 and S3 explants to 37.5% and 25.6%, respectively (Figure 2, LPNT). A combination of dipping in a 0.5 g·L^−1^ NaCl solution before planting and storing at 4 °C for 24 h after planting resulted in 10.0, 72.0, and 18.1% of the explants being at S1, S2, and S3 levels, respectively (Figure 2, NaLPNT).

Compared to those explants not transferred to a new medium, transferring the explants to a new medium was no longer effective in inhibiting explants to be brown at 4 weeks after culture initiation, especially when in combination with NaCl, low temperature, or both. Adding PVP to the medium was a very effective method to control the progress of explant browning, especially when combined with the NaCl solution dipping. Low temperature storage weakened PVP’s effectiveness of browning inhibition. Dipping in a NaCl solution before planting had a significant browning inhibitory effect, especially as combined with PVP addition. Furthermore, NaCl could adjust the native effect of low temperature storage. Hence, dipping in a NaCl solution before planting and PVP addition to the medium were the two most practical ways to inhibit browning and also to control the browning progress of the explants [21,36].

### 3.2. Effects of Treatments on Total Phenol Content and Enzyme Activities

**Figure 3:**
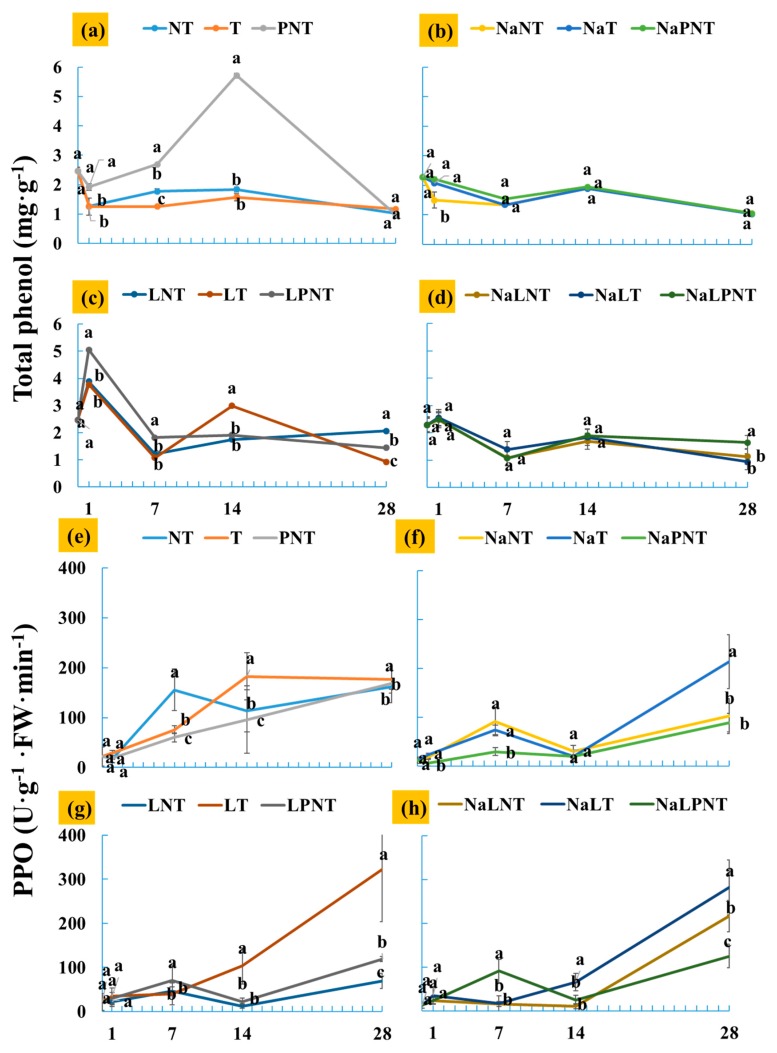

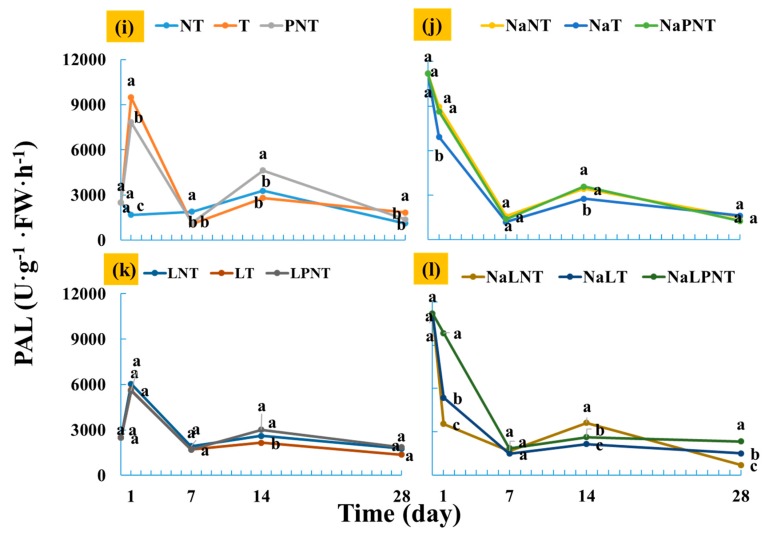
Total phenol contents (**a**–**d**), and activities of polyphenol oxidase (PPO) (**e**–**h**) and phenylalanine ammonia-lyase (PAL) (**i**–**l**) of peony petal explants. Refer to Figure 1 for details on the different denotations. Different alphabet letters indicate significant differences between treatments (ANOVA and Duncan’s test, *p* ≤ 0.05).

As shown in Figure 3, the total phenol content of the explants in different treatments showed different trends. When the petal explants were stored at 4 °C for 24 h after planting, the total phenol content increased gradually to 1 day after culture initiation (Figure 3c). This was consistent with the results in cauliflower heads [38] and fresh lettuce [35], but not with some outliers such as lotus root [39], which may be due to differences in the raw materials. Combining the NaCl dipping treatment with storage at 4 °C for 24 h after planting resulted in much lower total phenol contents of the explants than that of the explants without the NaCl solution dipping (Figure 3d). Different phenols are the main substrates for enzymatic browning in fruits and vegetables, and it seems that NaCl could inhibit the biosynthesis of total phenols [20].

Figure 3e–h shows the activities of enzyme PPO. It is obvious that transferring the explants to a new medium, dipping in a 0.5 g·L^−1^ NaCl solution before planting, storing at 4 °C for 24 h after planting, and adding PVP to the medium suppressed the PPO activity in the petal explants for the first two weeks. The PPO activity was the lowest in the explants when they were dipped in a 0.5 g·L^−1^ NaCl solution before planting, stored at 4 °C for 24 h after planting, and not transferred to a new medium. The PPO is a very important enzyme for the formation of the browning compound quinine [40,41]. Hence, transferring to a new medium, dipping in a 0.5 g·L^−1^ NaCl solution before planting, storing at 4 °C for 24 h after planting, and adding PVP to the medium are effective ways to delay explant browning by inhibiting the PPO activity.

The PAL is a key enzyme for the synthesis of phenolic compounds [42]. The PAL activities in the explants are presented in Figure 3i–l. The PAL activity increased to the peak at 0 day after culture initiation when the explants were dipped in a 0.5 g·L^−1^ NaCl solution before planting (Figure 3j,l). Without the NaCl solution dipping, the PAL activity peaked at 1 day after culture initiation (Figure 3I,k). Low temperature storage reduced the PAL activity regardless of treatment with NaCl solution dipping or PVP addition to the medium (Figure 3k,l). This is consistent with the results of PAL activity in pineapple [43] and lotus [39]. However, the PAL was found not to be significantly correlated to the PPO and total phenol contents in this study. There may be other enzymes involved in the metabolism [44].

### 3.3. Effects of Treatments on Explant Contamination and Callus Induction

**Figure 4:**
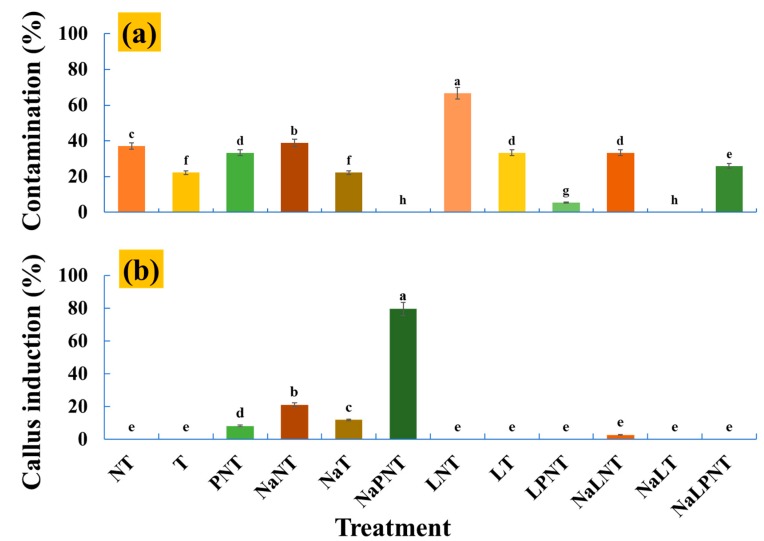
The percentage of explant contamination (**a**) and callus induction (**b**) of peony petal explants observed at 56 days after culture initiation. Refer to Figure 1 for details on the different denotations. Different alphabet letters indicate significant differences between treatments (ANOVA and Duncan’s test, *p* ≤ 0.05).

Figure 4a shows the contamination percentage of the explants observed at 8 weeks after culture initiation. The data clearly indicates that storage at 4 °C for 24 h after planting (LNT) had the greatest contamination percentage. Transferring the explants to a new medium may be a way to decrease the contamination percentage, because higher contamination percentages were recorded in the NT, NaNT, and NaLNT. As compared to the explants not transferred to new a medium, the contamination percentage of the explants in the T, NaT, LT, and NaLT was much lower. The PVP was reported as an effective browning inhibitor (Raghuvanshi and Srivastava 1995). In this study, adding PVP to the medium was also a good way to inhibit contamination, especially when it was combined with the NaCl solution dipping, since the lowest contamination percentage was observed in the NaPNT.

Callus induction is a key point of the success of many tissue cultures. The results of this study on callus induction are shown in Figure 4b. Dipping the explants in a 0.5 g·L^−1^ NaCl solution before planting significantly increased callus induction percentage. Adding PVP to the medium also enhanced callus induction. Combining PVP addition with dipping in a NaCl solution (NaPNT) gave the greatest callus induction percentage. However, when the explants were stored at 4 °C for 24 h after planting, the callus induction percentage decreased even along with NaCl solution dipping and PVP addition.

## 4. Conclusions

Browning is prevalent in tissue cultures of *Paeonia lactiflora* Pall. (herbaceous peony), and severely affects and restricts the growth and differentiation of the explants and/or success of tissue culture. In this study, dipping the excised explants in a sodium chloride (NaCl) solution as a pretreatment, adding polyvinyl pyrrolidone (PVP) to the medium, storing the planted explants at 4 °C for 24 h, and transferring planted explants to the same fresh medium after 24 h were all effective methods for suppressing browning of petal explants in tissue cultures of herbaceous peony ‘Festival Maxima’. It was observed in all treatments that the decreased browning was accompanied with higher levels of phenols and lower activities of phenylalanine ammonia-lyase (PAL) and polyphenoloxidase (PPO). Overall, the results suggest that dipping the explants in a NaCl solution was not only effective in suppressing browning of the petal explants of herbaceous peony, but also had positive synergistic effects with PVP added to the medium on callus induction.

## Figures and Tables

**Table 1:** The details on the treatment combinations.

Treatment Group	Treatment Number	Treatment Combination	Details of Treatment
a	1	NT	No transfer to a new medium
	2	T	Transfer to a new medium after 24 h of planting
	3	PNT	Addition of 0.5 g·L^−1^ PVP to the culture medium and no transfer to a new medium
b	4	NaNT	Dipping petals in a 0.5 g·L^−1^ NaCl solution before planting and no transfer to a new medium
	5	NaT	Dipping petals in a 0.5 g·L^−1^ NaCl solution before planting and transfer to a new medium after 24 h of planting
	6	NaPNT	Dipping petals in a 0.5 g·L^−1^ NaCl solution before planting, addition of 0.5 g·L^−1^ PVP to the culture medium, and no transfer to a new medium
c	7	LNT	Storage at 4 °C for 24 h after planting and no transfer to a new medium
	8	LT	Storage at 4 °C for 24 h after planting and transfer to a new medium after 24 h of planting
	9	LPNT	Storage at 4 °C for 24 h after planting, addition of 0.5 g·L^−1^ PVP to the culture medium, and no transfer to a new medium
d	10	NaLNT	Dipping petals in a 0.5 g·L^−1^ NaCl solution before planting, then storage at 4 °C for 24 h after planting, and no transfer to a new medium
	11	NaLT	Dipping petals in a 0.5 g·L^−1^ NaCl solution before planting, then storage at 4 °C for 24 h after planting, and transfer to a new medium after 24 h of planting
	12	NaLPNT	Dipping petals in a 0.5 g·L^−1^ NaCl solution before planting, then storage at 4 °C for 24 h after planting, addition of 0.5 g·L^−1^ PVP to the culture medium, and no transfer to a new medium after 24 h of planting
